# Special Issue “Centenarians—A Model to Study the Molecular Basis of Lifespan and Healthspan”

**DOI:** 10.3390/ijms22042044

**Published:** 2021-02-19

**Authors:** Calogero Caruso, Annibale Alessandro Puca

**Affiliations:** 1 Laboratory of Immunopathology and Immunosenescence, Department of Biomedicine, Neuroscience and Advanced Diagnostics, University of Palermo, 90134 Palermo, Italy; 2 Department of Medicine, Surgery and Dentistry “Scuola Medica Salernitana”, University of Salerno, 84081 Baronissi, Italy; apuca@unisa.it; 3 Cardiovascular Research Unit, IRCCS MultiMedica, 20138 Milan, Italy

## 1. State of Art

People are living longer, not, as was previously the case, due to reduced child mortality, but because we are postponing the ill-health of old age. The global population aged 60 years or over numbered 962 million in 2017, more than twice as large as in 1980 when there were 382 million older persons worldwide. The number of older persons is expected to double again by 2050, when it is projected to reach nearly 2.1 billion; the number of persons aged 80 years or over is projected to increase more than threefold between 2017 and 2050, rising from 137 million to 425 million. [1,2,3].

Ageing is a component of life, which derives from the breakdown of the self-organization system and the reduced ability to adapt to the environment. As we age, harmful changes accumulate in the molecules, cells and tissues responsible for the decline of normal physiological functions. This inexorably leads to a reduced ability of the individual to maintain adequate homeostasis, resulting in a greater susceptibility to different types of stressors. Hence, ageing processes are defined as those that amplify the vulnerability of individuals, as they age, to the factors that ultimately lead to death [4].

This extraordinary increase in older people emphasizes the importance of studies on ageing and longevity and the need for a prompt dissemination of knowledge on ageing and longevity with the aim to satisfactorily diminish the medical, economic and social problems associated with advancing years, problems caused by the continuous increase in the number of older people at risk of frailty and age-related diseases. In fact, the increase in the duration of life (lifespan) does not coincide with the increase in the duration of health (healthspan), that is the period of life free from serious chronic diseases and disabilities [1,5]. Therefore, improving the quality of life of older people must be a priority. This makes studies of the processes involved in ageing and longevity of great importance.

In particular, understanding why some individuals have escaped from neonatal mortality, infectious diseases in the pre-antibiotic era and the fatal outcomes of age-related diseases, thus living 100+ years, might allow the factors involved in the attainment of healthy ageing. For scientists, centenarians are, in fact, the paradigmatic example of healthy ageing [6].

Long-lived individuals should refer to people belonging to the 5th percentile of the survival curve, that is, in the Western world, to those over ninety. However, it is often the canonical age of 100 that is regarded as the threshold of exceptional longevity [4,7]. Worldwide, the number of centenarians fluctuates between half a million and a million with five women for one man. Over the course of human history their number has grown [2,8].

Human beings are the product of their genes, but they would not be the same without the experiences they have/had since those in the womb. It has been shown that the month of birth, a proxy for early environmental influences (i.e., epigenetics), affects the possibility of reaching 100 years [9]. Living organisms are subject to nature laws and genetic programs where both Brownian random motions, i.e., the erratic random movement of particles in a fluid, as a result of continuous bombardment from molecules of the surrounding water molecules in the fluid, and crossing over contribute to leave space for the chance. There is evidence of the inherent stochastic nature of both gene expression and macromolecular biosynthesis. Several genes are in fact transcribed in minimal amounts of mRNA, which can cause large fluctuations in macromolecular biosynthesis. Chance is just that, the random occurrence, i.e., an event happening not according to a plan [2,10].

Asking whether ageing and longevity depend on the environment or genetics, even if legitimate, is too simplified. To respond fully means to consider all that is important, the case and circumstances of life, diet, physical activity, environmental exposure and lifestyle (which also affect epigenetics), the burden of natural life pathogens, stress management and social networking, education and gender and, obviously, DNA (genetics and epigenetics) in the forefront [10,11,12,13]. However, taking into account the risk factors we can modify lifestyle, particularly diet, that is, beyond doubt, the most important.

The deepening of this knowledge could allow modulating the ageing rate by providing valuable information on how to achieve healthy ageing.

## 2. Diet and Healthy Ageing

A long life in a healthy, vigorous and young body has always been one of humanity’s greatest dreams. Anti-ageing strategies aimed not at rejuvenating but at slowing ageing and delaying or avoiding the onset of age-related diseases are welcome. It has to be emphasized that the goal of ageing research is not to increase human longevity regardless of the consequences, but to increase active life free from disability and functional dependence.

As previously stated, the role of diet in the attainment of longevity by slowing ageing and fighting age-related diseases is well known. A particular aspect of diet concerns some compounds principally contained in fruit and vegetables, called nutraceuticals, i.e., “naturally derived bioactive compounds that are found in foods, dietary supplements and herbal products, and have health promoting, disease preventing, and/or medicinal properties”. Several nutraceuticals exhibit anti-ageing features by acting on the inflammatory status and on the prevention of oxidative reaction. Similar effects are thought to be obtained with the use of probiotics [14,15]. In this special issue six papers addressed these aspects in vitro or in vivo.

In an in vitro study [16], it was examined in pheochromocytoma 12 (PC12) cells, whether *Hericium erinaceus* (HE), a medicinal plant, could exert a protective effect against oxidative stress and apoptosis induced by di(2-ethylhexyl)phthalate (DEHP), a plasticizer known to cause neurotoxicity. The authors demonstrated that pre-treatment with HE significantly attenuated DEHP induced cell death. This protective effect was attributed to its ability to reduce intracellular reactive oxygen species levels.

Two studies [17,18] focused on the role played by some olive oil polyphenols [19] on ageing pathophysiology with special attention to Parkinson’s disease like symptoms in the well-known invertebrate model organism *Caenorhabditis (C) elegans*. The studies demonstrate that polyphenolic extract treatment has the potential to partly prevent or even treat ageing-related neurodegenerative diseases and ageing itself. Future investigations including mammalian models and human clinical trials are needed to uncover the full potential of these olive compounds.

The review of Rosselli et al., [20], analyzed, instead, the impact of probiotics on *C. elegans.* The picture emerging from their analysis highlights that several probiotic strains are able to exert anti-ageing effects in nematodes by acting on common molecular pathways, such as insulin/insulin-like growth factor-1 and p38 mitogen-activated protein kinase. So, *C. elegans* appears to be advantageous for shedding light on key mechanisms involved in host pro-longevity in response to probiotics supplementation.

Then, in their review [21], the authors discuss the possible effect of Royal Jelly and its components on healthy ageing and longevity in animal models as well as its positive effects on health maintenance and age-related disorders in humans. The findings pave the way to inventing specific Royal Jelly as anti-ageing drugs.

Finally, a small clinical trial was instead performed on another model of neurodegeneration, Meniere’s disease (MD) [22]. The authors evaluated systemic oxidative stress and cellular stress response in MD patients in the absence and in the presence of treatment with a biomass preparation from *Coriolus versicolor*, endowed with various biological actions, including antioxidant ones. It was concluded that systemic oxidative stress was reduced in MD patients treated with *Coriolus versicolor*, which was paralleled by a significant induction of vitagenes, known to encode survival and anti-oxidant proteins. Vitagene up-regulation after *Coriolus versicolor* supplementation indicates a maintained response to counteract intracellular pro-oxidant status. Thus, searching innovative and more potent inducers of the vitagene system can allow the development of pharmacological strategies capable of enhancing the intrinsic reserve of vulnerable neurons, such as ganglion cells to maximize antidegenerative stress responses and thus providing neuroprotection. 

## 3. Hallmarks of Ageing

Some aspects fighting three hallmarks of ageing, i.e., characteristic features considered to contribute to the ageing process, hence determining the ageing phenotype [23], are instead treated in the last four papers.

Concerning the hallmark altered intercellular communication [23], e.g., in this series, Adverse Food Reactions (FAs) [24] have been treated as example of immunosenescence [25]. FAs show peculiar characteristics in older people that concern both the pathogenesis and the clinic. FAs in older people are driven by immunosenescence, as well as the cell ageing and tissue modifications that characterize advanced age. The aged gastrointestinal mucosa is central in the development of FAs in older people through its compromised digestive properties and structural changes, as well as the alteration of its immune functions linked to immunosenescence and age-related microbiota remodeling. Among the risk factors for the sensitization to food allergens in older people, in addition to chronic damage and inflammation of gut epithelia due to the ageing process, there are chronic alcohol consumption, chronic infections, multimorbidity, polymedication, and drug side effects.

Data of Jimenez-Gutierrez et al., [26] regard the prevention of the hallmark cellular senescence [23]. Their data are consistent with the paradigm that interfering with function of Nuclear β-dystroglycan (β-DG) involved in the maintenance of nuclear architecture and function results somehow in aberrant multipolar mitoses. That, in turn, evokes a p53-dependent DNA-damage response, arresting the cell cycle progression and thereby inducing senescence, to avoid the propagation of damaged genomes. That supports a role for DG in protecting against senescence, through the maintenance of proper lamin B1 expression/localization and proper mitotic spindle organization.

Epigenetics, another hallmark [23] is treated in the last two papers. In the first paper [27], the authors used next-generation sequencing to identify differentially expressed microRNAs (miRNAs) in serum exosomes isolated from young (three-month-old) and old (22-month-old) rats and then used bioinformatics to explore candidate genes and ageing-related pathways. Taken together, their findings suggest that changes in the makeup of circulating exosomal miRNAs with age not only can be considered as a potential predictor of age but also may contribute to ageing via several key signaling pathways that regulate ageing and lifespan.

In the paper of Gutman et al. [28], exceptionally long-lived individuals (ELLIs) demonstrated juvenile performance in DNA methylation age clocks and overall methylation measurement, with preserved cognition and relative telomere length. The findings suggest a favorable DNA methylation profile in ELLIs enabling a slower rate of ageing in those individuals in comparison to controls. It is possible that DNA methylation is a key modulator of the rate of ageing and thus the ELLIs DNAm profile promotes healthy longevity.

## 4. Conclusions

This series of papers is noteworthy because the identification of the factors that predispose to a long and healthy life is of enormous interest for translational medicine.
